# The Use of Tobacco

**Published:** 1881-08

**Authors:** 


### SELECTIONS.

### THE USE OF TOBACCO.

On this subject the editor of the *Medical and Surgical Reporter* in a recent number, says:

"The forms in which it is used have come in for a share of the discussion. One writer was of opinion that smoking a pipe is the least injurious, which is certainly contrary to the general opinion. The cigar, at any rate, is rapidly supplanting the pipe in civilized society. Snuffing, which was so fashionable a hundred years ago, and was probably less hurtful than smoking, has gone out of date in the better circles.

"The whole subject is evidently one which could be advantageously studied again, provided the investigator divested himself of all partiality for or prejudice against the habit. For a long time we have had no report on the weed, of the author of which this can be said.

"If we are willing to accept the opinions which sanitarians in other nations have formed, we have a very decided one ready to our hand in Switzerland. That intelligent republic enacted a law, last year, prohibiting the sale of tobacco to minors under fifteen years of age, and making it an offense against the law for such to smoke. Hence a boy of twelve or fourteen who parades the streets of Geneva or Bern with a cigar in his mouth is liable to be arrested and committed to the police station; and as they have a disagreeable habit in that republic of enforcing the laws they enact, such would be pretty certain to be the juvenile smoker's fate.

"We recommend to our fellow-countrymen their manner of dealing with the habit, which, whether harmless or not to most adults, is unquestionably of great injury to young boys."

